# Supplementary material for: The interactome of intact mitochondria by cross-linking mass spectrometry provides evidence for coexisting respiratory supercomplexes
Source: Mol Cell Proteomics. 2017 Dec 8;17(2):216–32. doi: 10.1074/mcp.RA117.000470 (PMC5795388; doi:10.1074/mcp.RA117.000470)
Supplement: Supplemental Data [file supp_RA117.000470_133922_0_supp_23978_fzffwf.zip › spectra_annotation/mito_DR_spectra_annotation/173-1-1-1-188-1.pdf]

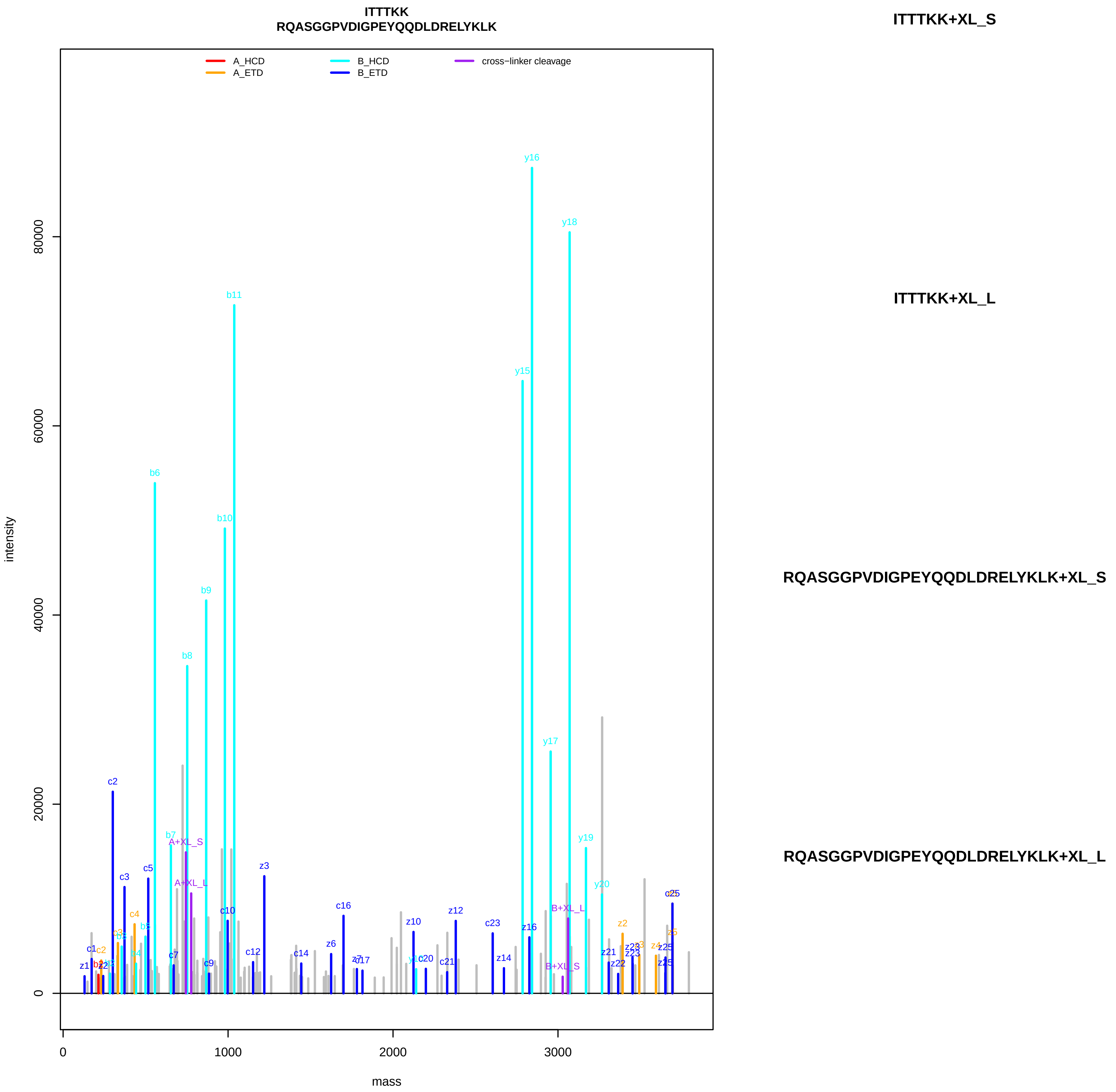
ITTTKK
RQASGGPVDIGPEYQQDLDRELYKLK
A_HCD
A_ETD
B_HCD
B_ETD
cross−linker cleavage
intensity
mass
0
20000
40000
60000
80000
0
1000
2000
3000
y16
y18
b11
y15
b6
b10
b9
b8
y17
c2
b7
A+XL_S
y19
c3
c5
z3
A+XL_L
y20
c25
c10
c16
B+XL_L
z12
z10
c23
c4
z16
z2
c1
c2
c7
c12
c14
z6
c20
c21
z14
z21
z22
z23
z25
B+XL_S
c9
z1
z4
ITTTKK+XL_S
ITTTKK+XL_L
RQASGGPVDIGPEYQQDLDRELYKLK+XL_S
RQASGGPVDIGPEYQQDLDRELYKLK+XL_L
